# Cardiac conduction system malformations in heterotaxy result from dysregulated *Pitx2* expression

**DOI:** 10.1172/jci.insight.199072

**Published:** 2026-02-24

**Authors:** Kunihiko Joo, Ryohei Matsuoka, Keiko Kitajima, Kenta Yashiro, Akira Shiose, Ryuji Tominaga, Michael M. Shen, Shinya Oki, Chikara Meno

**Affiliations:** 1 Department of Developmental Biology,; 2 Department of Cardiovascular Surgery, and; 3 Department of Pediatrics, Graduate School of Medical Sciences, Kyushu University, Fukuoka, Japan.; 4 Department of Anatomy, Kyoto Prefectural University of Medicine, Kyoto, Japan.; 5 Department of Medicine, Columbia University Irving Medical Center, New York, New York, USA.

**Keywords:** Cardiology, Development, Cardiovascular disease, Embryonic development

## Abstract

The cardiac conduction system (CCS) develops asymmetrically along the body axes. In heterotaxy syndrome — resulting from aberrant left-right axis formation — atrial and atrioventricular conduction defects can cause life-threatening arrhythmias. However, the developmental mechanisms regulating the atrioventricular conduction system (AVCS) disposition and integrity remain unclear. To investigate the etiology of AVCS malformations in laterality defects, we analyzed CCS development and function in mouse mutants for *Cryptic* and *Lefty1*, which are key regulators of *Pitx2* in the left-right axis formation. *Cryptic*^–/–^ embryos exhibited bilateral sinoatrial nodes and an ectopic anterior AV node and bundle accompanied by reduced *Pitx2* expression. In contrast, *Lefty1*^–/–^ embryos showed a hypoplastic sinoatrial node and AV node–bundle dissociation with ectopic *Pitx2* expression. Single-cell transcriptomic analysis of *Pitx2*^–/–^ hearts revealed expansion of AV node and bundle populations, consistent with a repressive role of *Pitx2* in AVCS specification. Genetic lineage tracing indicated that *Pitx2*-expressing cells from the left lateral plate mesoderm populate cranioventral cardiac regions, where AVCS development is suppressed. Together, these findings clarify how global left-right axis information is locally integrated to shape AVCS disposition and integrity, providing a mechanistic model for AVCS abnormalities in laterality-associated congenital heart disease.

## Abstract 2

Although the atrioventricular conduction pathway is known, how its placement and integrity are organized was unclear. We show it depends on left–right body axis.

## Graphical abstract

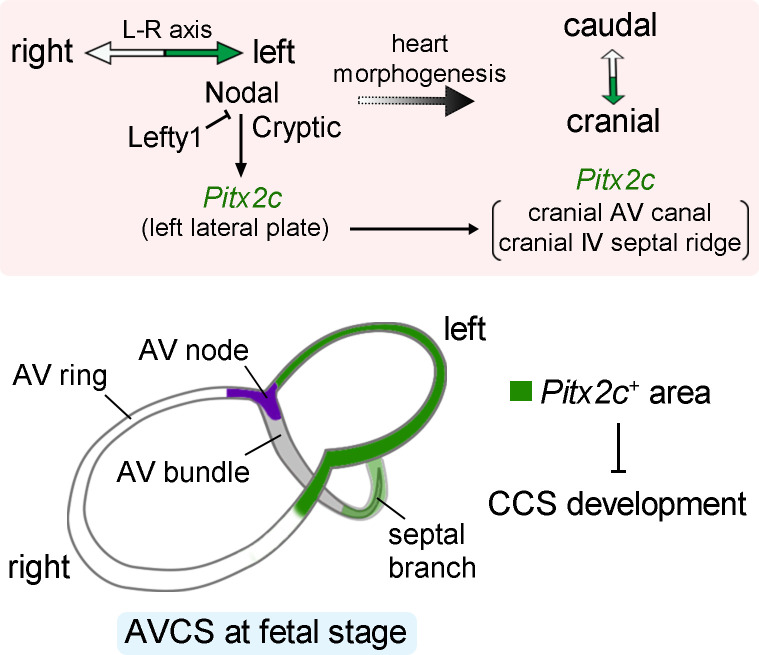

## Introduction

The rhythmic and coordinated contraction of the heart is enabled by the cardiac conduction system (CCS) (1, 2). It comprises specialized cardiomyocytes, including the sinoatrial (SA) node, atrioventricular (AV) node, AV bundle, bundle branches, and Purkinje fibers. The SA node, located at the junction of the superior caval vein and the right atrium, functions as the pacemaker. Slow diastolic depolarization mediated by hyperpolarization-activated cyclic nucleotide-gated potassium channel 4 (Hcn4) at the SA node leads to the generation of an action potential that propagates to trigger atrial contraction (3, 4). With electrical insulation provided by the fibrous skeleton at the base of the ventricles, the AV node — situated posteriorly at the base of the interatrial septum — delays the transmission of the electrical impulse, allowing sufficient time for ventricular filling. The ventricular conduction pathway distal to the AV node rapidly transmits the impulse, resulting in synchronized ventricular contraction and blood ejection into the systemic or pulmonary circulation.

Two additional specialized tissues extend from the AV conduction pathway: the septal branch (also known as the dead-end tract or aortic ring), which emerges from the AV bundle and courses cranially around the base of the aorta (5–7), and the AV rings, extending from the AV node and surrounding the AV orifices above the fibrous annulus (5, 8, 9). Because of their essential role in heart contraction, disorders and malformations of CCS components cause arrhythmias, potentially leading to syncope, heart failure, or sudden death (10). For instance, many patients with heterotaxy syndrome experience arrhythmias, such as supraventricular tachycardia in right isomerism or sinus bradycardia/AV block in left isomerism (11).

The AV conduction system (AVCS) derives from the embryonic AV canal and the interventricular (IV) ring in the IV septal ridge, which give rise to the compact AV node and lower nodal cells/AV bundle, respectively (5). The AV node develops at the intersection of the inferior AV canal and the caudal IV ring, whereas the septal branch derived from the cranial IV ring loses its connection with the AV canal–derived AV ring (5, 12). Transcriptional networks govern the formation and function of each CCS component (1, 2). Among them, the T-box transcription factor Tbx3 plays a pivotal role by suppressing the gene program for working myocardium (12–16). In embryonic hearts, *Tbx3* is expressed in the AV canal, IV ring, and SA node; therefore, these expression domains delineate the developing central CCS, similar to *Hcn4* (3, 12, 17). Formation of the SA node is regulated by *Pitx2*, which encodes a paired-like homeodomain transcription factor essential for heart morphogenesis (18). Left-sided expression of *Pitx2* suppresses *Tbx3* and *Shox2* expression, thereby restricting SA node formation to the right side (19–24).

*Pitx2* expression in the developing heart is primarily defined by the left-right axis that is established before the formation of the heart tube (25–29). The left-right axis is generated in the lateral plate mesoderm (LPM) through autoregulatory loops of *Nodal* and *Lefty* genes, which encode members of the TGF-β superfamily (28). *Nodal* is expressed in the left LPM, where it signals through a forkhead DNA-binding transcription factor, Foxh1. An intronic *Pitx2* enhancer, known as *ASE*, contains binding sites for Foxh1, Nkx2-5, and Tbx1, through which transcription of the *Pitx2c* isoform is induced by Nodal in the left LPM and maintained in its derivatives, including the heart (25, 30, 31). Cryptic (Cfc1), an EGF-CFC family member, functions as a coreceptor for Nodal and is required for Nodal signaling in the LPM. In *Cryptic*^–/–^ embryos, failure to induce *Pitx2c* expression in the left LPM leads to laterality defects characterized by right isomerism (29, 32, 33). In contrast, Lefty restricts Nodal activity to the left side. Thus, the loss of *Lefty1* causes left isomerism via bilateral expression of *Pitx2* (26). Despite advances in understanding of the left-right axis and detailed descriptions of the CCS in human heterotaxy syndrome (34–37), experimental studies examining CCS development in mouse models of left-right axis defects remain limited (24, 36, 38–40).

Here, we examined CCS formation in *Cryptic* and *Lefty1* mutant embryos. In addition to aberrant SA node formation, both mutants showed altered AVCS patterning, consistent with the features of human heterotaxy syndrome. Our findings suggest that the left-right axis plays a decisive role in shaping the asymmetric disposition of the AVCS, probably via Pitx2-dependent local suppression of CCS formation, providing a mechanistic basis for conduction defects in congenital laterality disorders.

## Results

### Disposition of CCS in Cryptic^–/–^ embryos at E18.5.

To investigate CCS disposition in mouse heterotaxy, we first examined the hearts of *Cryptic*^–/–^ embryos at E18.5, which develop right isomerism (32, 33). Although cardiac looping is reported to be randomized in *Cryptic*^–/–^ mice (32), embryos carrying the same mutation on an FVB/N background showed a d-loop (*n* = 7/7 at E18.5; *n* = 8/8 at E14.5; *n* = 6/6 at E12.5), indicating that the looping phenotype depends on the genetic background. Characteristic congenital heart diseases (CHDs) included common atrium (*n* = 7/7), atrioventricular septal defect (AVSD) (*n* = 7/7), transposition of the great arteries (TGA) (*n* = 5/7), double outlet right ventricle (DORV) (*n* = 2/7), and total anomalous pulmonary venous connection (TAPVC) (*n* = 7/7) — features typically observed in human right atrial isomerism (34) (see Supplemental Table 1 for full description of mutant hearts; supplemental material available online with this article; https://doi.org/10.1172/jci.insight.199072DS1).

**Figure 1:**
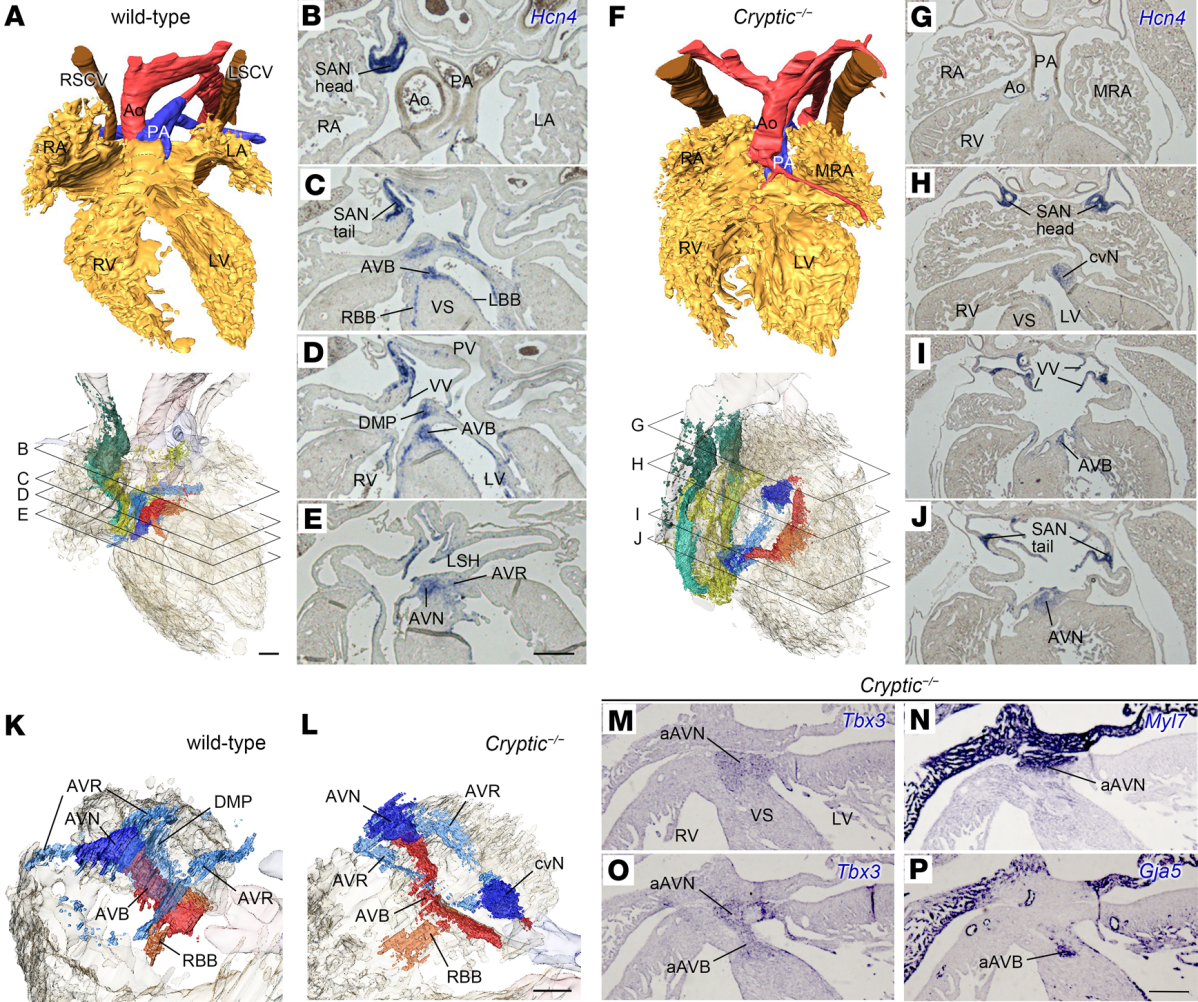
Abnormal cardiac conduction system in the hearts of *Cryptic*^–/–^ embryos at E18.5. (**A**–**J**) Representative images of 3D-reconstructed hearts (**A** and **F**) and their original images of in situ hybridization with *Hcn4* probes (**B**–**E** and **G**–**J**) in WT (**A**–**E**) (*n* = 6) and *Cryptic*^–/–^ (**F**–**J**) embryos (*n* = 4) at E18.5. Upper panels in **A** and **F** are ventral views showing the lumen of heart chambers and great vessels, whereas lower panels are right ventral views showing *Hcn4* expression in pseudocolors (SA node head, dark green; SA node tail, pale green; venous valves and sinus horn, yellow; AV ring, light blue; AV node and cranioventral nodule, blue; AV bundle and septal branch, red; bundle branches, orange). The levels for each transverse section are shown in the lower panels. (**K** and **L**) Right craniodorsal views of the base showing *Hcn4* expression in the WT (**K**) and *Cryptic*^–/–^ (**L**) hearts presented in **A** and **F**, respectively. (**M**–**P**) Expression of *Tbx3* (**M** and **O**), *Myl7* (**N**), and *Gja5* (**P**) was detected in adjacent transverse sections of a *Cryptic*^–/–^ heart (different from **F**), corresponding to the level shown in **H**. Scale bars: 200 μm. aAVB, (anterior) atrioventricular bundle; aAVN, (anterior) atrioventricular node; Ao, aorta; AVR, atrioventricular ring; cvN, cranioventral nodule; DMP, dorsal mesenchymal protrusion–derived tissue; LA, left atrium; (L/R)BB, (left/right) bundle branch; (L/R)SCV, (left/right) superior caval vein; LSH, left sinus horn; LV, left ventricle; (M)RA, (morphologically) right atrium; PA, pulmonary artery; PV, pulmonary vein; RV, right ventricle; SAN, sinoatrial node; VS, ventricular septum; VV, venous valve.

Specialized cardiomyocytes were detected using in situ hybridization of *Hcn4*, followed by 3D reconstruction of the CCS (WT, *n* = 6; *Cryptic*^–/–^, *n* = 4) (Figure 1, A–L). In *Cryptic*^–/–^ embryos, bilateral SA nodes were present, albeit with smaller sizes compared with that in the WT embryos (*n* = 4/4) (Figure 1, B and H). Although the AV node was normally connected to the AV bundle, its position had shifted caudally (Figure 1, A and F). Left and right bundle branches were identified as continuations of the AV bundle, retaining their characteristic morphologies (Supplemental Figure 1). Notably, a distinct, intensively *Hcn4*-expressing nodule was detected in the ventricle, continuous with the AV ring at the cranioventral position (*n* = 4/4) (Figure 1, H and L). Immunostaining confirmed the presence of Hcn4 protein in the SA nodes and cranioventral nodule of the *Cryptic*^–/–^ heart, consistent with the in situ hybridization results (Supplemental Figure 2). In humans, right atrial isomerism is accompanied by bilateral SA nodes and dual AV nodes that often connect with the AV bundles to form the sling (34, 35, 37). To confirm the identity of the *Hcn4*^+^ nodule as an anterior AV node, we examined additional markers — *Tbx3* and *Myl7* for the AV node (12, 38) and *Tbx3* and *Gja5* for the AV bundle (12, 41) — in 3 *Cryptic*^–/–^ hearts. The nodule was *Tbx3*^+^/*Myl7*^+^ (*n* = 3/3) (Figure 1, M and N, with the control shown in Supplemental Figure 3), supporting its identification as an AV node. In addition, the sling was observed in one heart in which continuous expression of *Tbx3* in both the anterior AV node and the anterior AV bundle (*Gja5*^+^) was detected (*n* = 1/3) (Figure 1, O and P). These results indicate that the left-right axis shapes the disposition of the AV conduction system.

### CCS development in Cryptic^–/–^ embryos.

**Figure 2:**
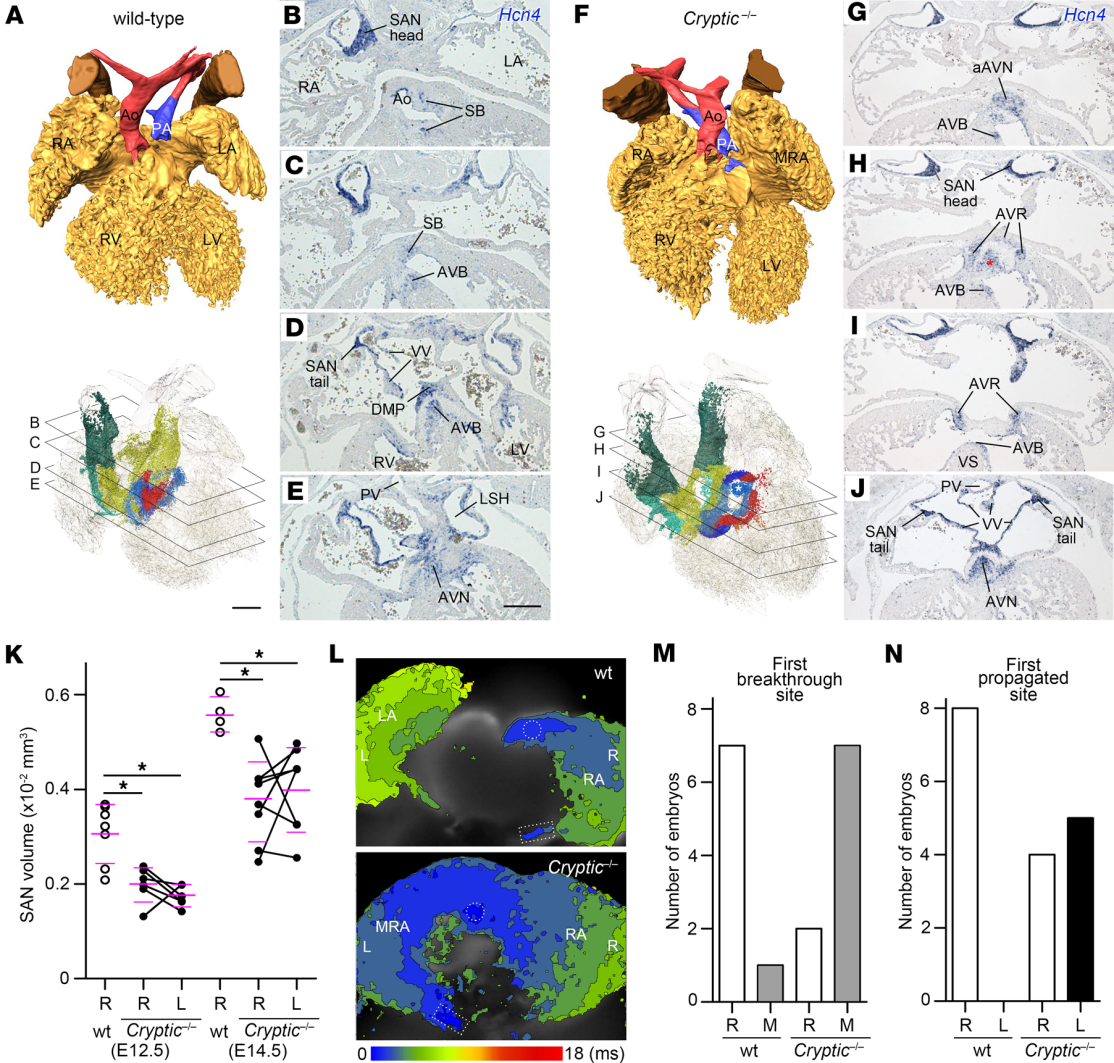
Abnormal cardiac conduction system development in the hearts of *Cryptic*^–/–^ embryos at E12.5–E14.5. (**A**–**J)** Representative images of 3D-reconstructed hearts (**A** and **F**) and their original images of in situ hybridization with *Hcn4* probes (**B**–**E** and **G**–**J**) in WT (**A**–**E**) (*n* = 8) and *Cryptic*^–/–^ (**F**–**J**) embryos (*n* = 8) at E14.5, as presented in Figure 1, A–E. The asterisks in **F** and **H** show *Hcn4* expression in the superior cushion. Scale bars: 200 μm. (**K**) SA node head volumes of WT and *Cryptic*^–/–^ embryos at E12.5 (*n* = 6–7; biological replicates) and E14.5 (*n* = 4–7; biological replicates). The left and right values of the same embryo are connected. The magenta bars represent mean ± SD. *P < 0.01 (1-way ANOVA followed by Dunnett’s multiple-comparison test). (**L**) Representative images of voltage mapping with Di-4-ANEPPS in WT (*n* = 8) and *Cryptic*^–/–^ hearts (*n* = 9) at E12.5 (dorsal views). The dotted circles and rectangles indicate the sites where action potential first appeared, around the head and tail of the SA node, respectively. A single heartbeat is shown. (**M** and **N**) First breakthrough site of the action potential around the SA node head (**M**) and atrial side where the action potential first propagated (**N**). Quantification is based on the final heartbeat from each heart (*n* = 8–9; biological replicates). Abbreviations are the same as in Figure 1, except for L, left; M, middle; R, right; SB, septal branch.

To elucidate the process of abnormal CCS formation in *Cryptic*^–/–^ embryos, we examined *Hcn4* expression at E12.5–E14.5. In the WT atrium at these stages, *Hcn4* was continuously expressed in the SA node, venous valves, and dorsal mesenchymal protrusion–derived tissue (42, 43) (Figure 2, A–E, and Supplemental Figure 4, A and B). AV canal–derived tissues, including the AV node, AV rings, and forming AV valves, also expressed *Hcn4* (3, 5, 17) (Figure 2, A, D, and E, and Supplemental Figure 4, B and C). Additionally, *Hcn4* expression was evident in the caudal IV ring/AV bundle at E12.5 and E14.5 (17) (Figure 2, A, C, and D, and Supplemental Figure 4B). The septal branch derived from the cranial IV ring was faintly and discontinuously detected with the *Hcn4* probe at E14.5, but was undetectable at E12.5*,* whereas an *Hcn4*-positive cell population was observed in the subaortic portion at E14.5, possibly representing part of the septal branch near the AV ring (Figure 2, A–C, and Supplemental Figure 4A).

In *Cryptic*^–/–^ embryos, the SA nodes were bilaterally developed (*n* = 8/8 at E14.5; *n* = 6/6 at E12.5), from which the contiguous expression of *Hcn4* extended to the venous valves on the left and right sides (Figure 2, F–J, and Supplemental Figure 4, D and E). Dorsal mesenchymal protrusion formation was markedly impaired (Figure 2I and Supplemental Figure 4E), probably accounting for the development of AVSD (44). Although *Hcn4*-positive AV rings were detected at E14.5, their cranioventral portions were dorsally bent (Figure 2, F and H). In the superior AV cushion-derived tissue, a distinct *Hcn4*-positive domain formed a fermata-like mark with the bent AV rings in the transverse sections (*n* = 7/8) (Figure 2H, asterisk) that continued caudally and tapered into the cushion. The prominent *Hcn4*-expressing nodule was detected as a protrusion of the cranioventral AV ring at E14.5 (*n* = 8/8) (Figure 2G). In one heart at E14.5, the cranioventral nodule was apparently continuous with the *Hcn4*-positive bundle (Supplemental Figure 4F), whereas in others, discontinuous *Hcn4* expression in the septal branch was detected (*n* = 7/8), indicating that a part of *Cryptic*^–/–^ embryos developed the anterior AV bundle instead of the septal branch.

To evaluate the development and function of the SA node, we first measured the SA node volume using 3D reconstruction of the *Hcn4* in situ hybridization sections. The volumes were similar on both sides in *Cryptic*^–/–^ embryos at E12.5 and E14.5, and were significantly smaller than those in WT embryos (Figure 2K). Cardiac voltage mapping of isolated hearts at E12.5 detected the appearance of action potentials around the SA node, in which simultaneous firing around the head and tail of the SA node (13) was observed in WT (16/36 beats in 8 hearts) and *Cryptic*^–/–^ (19/37 beats in 9 hearts) hearts (Figure 2L). The first breakthrough site in the craniodorsal portion of the atria was located on the right (*n* = 7/8) or middle (*n* = 1/8) of WT hearts, whereas the middle site was significantly more frequent in *Cryptic*^–/–^ hearts (right: *n* = 2/9; middle: *n* = 7/9; *P* = 0.0152, Fisher’s exact test) (Figure 2M). In addition, while the right atrium propagated action potential earlier than the left atrium in WT hearts (*n* = 8/8), the first propagated side of the atrium was either the right (*n* = 4/9) or left (*n* = 5/9) in *Cryptic*^–/–^ hearts (*P* = 0.0294, Fisher’s exact test) (Figure 2N). These changes in the pacemaker position and action potential propagation likely result from bilaterally formed SA nodes, as has been reported for *Pitx2* mutant embryos (24, 40). We also recorded successive atrial and ventricular action potential traces in *Cryptic*^–/–^ hearts at E12.5 by voltage mapping but did not observe any obvious abnormalities compared with WT hearts (Supplemental Figure 5).

### Relationship between Pitx2 expression domains and CCS.

**Figure 3:**
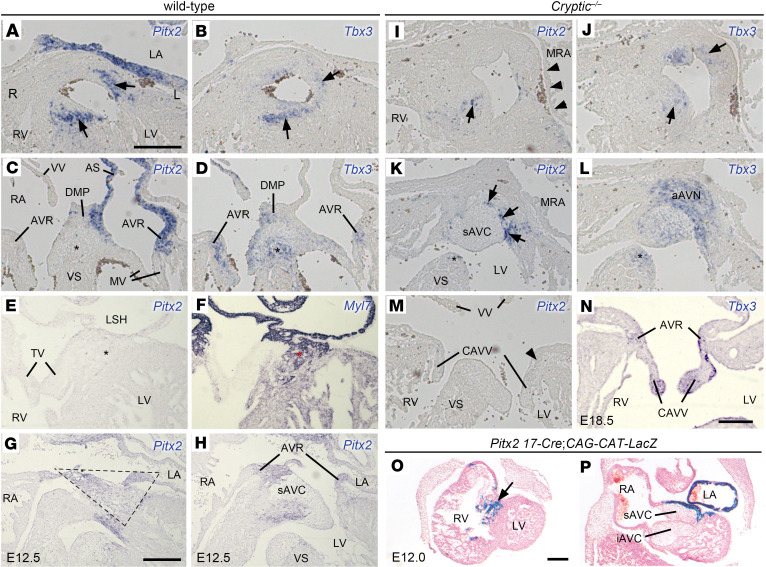
*Pitx2* expression domains in the cardiac conduction system and its cell lineages. (**A**–**N**) Transverse sections of hearts from WT (**A**–**H**) and *Cryptic*^–/–^ (**I**–**N**) embryos stained by in situ hybridization at E14.5 (**A**–**F** and **I**–**M**), E12.5 (**G** and **H**), and E18.5 (**N**) for *Pitx2* (**A**, **C**, **E**, **G**–**I**, **K**, and **M**), *Tbx3* (**B**, **D**, **J**, **L**, and **N**), and *Myl7* (**F**). The arrows indicate *Pitx2* or *Tbx3* expression in the septal branch (**A**, **B**, **I**, and **J**) and in the cranioventral side of the AV rings (**K**). The asterisks in **C**, **D**, **K**, and **L** indicate the AV bundle, whereas those in **E** and **F** denote the AV node. The arrowheads indicate loss of *Pitx2* expression in the atrium (**I**) and left AV ring (**M**). The dashed rectangle in **G** indicates *Pitx2*^+^ superior AV canal myocardium, including AV rings. The arrows in **I** and **K** show residual expression of *Pitx2* in the septal branch and AV rings, respectively. (**O** and **P**) X-gal staining of a *Pitx2 17-Cre* *CAG-CAT-LacZ* heart at E12.0. Coronal sections are shown. The arrow indicates the septal branch. Scale bars: 200 μm. The scale bars in **A**, **G**, and **O** apply to **A**–**F** and **I**–**M**, **G** and **H**, and **O** and **P**, respectively. Abbreviations are the same as in Figure 1, except for AS, atrial septum; CAVV, common atrioventricular valve; (i/s)AVC, (inferior/superior) atrioventricular cushion; L, left; MV, mitral valve; R, right; TV, tricuspid valve.

Because *Cryptic* is necessary for inducing *Pitx2c* expression in the left LPM (32, 33), the loss of *Pitx2c* expression in the heart likely underlies the abnormal CCS in *Cryptic*^–/–^ embryos. To understand the relationship between CCS-related genes and *Pitx2*, we compared their expression domains in adjacent sections of WT and *Cryptic*^–/–^ hearts at E14.5. In WT embryos, *Shox2*, *Tbx3*, and *Hcn4* colocalized in the SA node and venous valves, which lack *Pitx2* expression (Supplemental Figure 6) (3, 12, 19, 20). In *Cryptic*^–/–^ embryos, *Pitx2* expression in the left atrium, left superior caval vein, and pulmonary veins was lost, as expected (Figure 3, A, C, and I–K, and Supplemental Figure 6), whereas *Tbx3* and *Shox2* were bilaterally expressed in the SA nodes, similar to *Hcn4* (Supplemental Figure 6). Notably, *Shox2* expression in the pulmonary veins (20) was downregulated, probably because of the absence of a myocardial sleeve, as indicated by the lack of *Myl7* expression accompanied by the loss of *Pitx2* expression (Supplemental Figure 6) (45).

*Pitx2* is expressed in the superior/left lateral AV canal and septal branch (25, 27, 29, 46). Concordantly, *Pitx2* expression in WT hearts at E14.5 was apparent in the left AV ring and septal branch (Figure 3, A and C), which are the regions where *Tbx3* and *Hcn4* expression was later downregulated (Figure 2, A–D, and Figure 3, B and D) (8, 12). In contrast, *Pitx2* expression was not detected in the AV node and AV bundle (Figure 3, C and E, and Figure 3, D and F, using *Tbx3* and *Myl7* as CCS markers). At E12.5, the cranioventral portion of the AV rings and cardiomyocytes in the superior AV cushion were *Pitx2-*positive (Figure 3, G and H), which corresponded to the regions with upregulated *Hcn4* expression in *Cryptic*^–/–^ embryos (Figure 2, F–H). Unexpectedly, in *Cryptic*^–/–^ embryos, *Pitx2* expression was detected in the septal branch at a reduced level (Figure 3, I and J, using *Tbx3* as a CCS marker). *Pitx2* expression was nearly absent in the left AV ring (Figure 3M), whereas it was sporadically detected in the cranioventral portion of the AV rings (Figure 3K). Consistent with the diminished expression of *Pitx2* at E14.5, extensive *Tbx3* expression was observed in the anterior AV node and left AV ring of *Cryptic*^–/–^ embryos (Figure 1M and Figure 3, L and N) (23). Because the superior and left-lateral AV canal is derived from the left second heart field (27, 29, 47), we traced the descendants of *Pitx2*-expressing cells in the left LPM using the *Pitx2 17*-*Cre* transgene, whose enhancer activity depends on *Pitx2 ASE* regulated by the Nodal signal in the left LPM (29). Embryos from crosses with *CAG-CAT-LacZ* mice showed X-gal staining in the septal branch and superior AV canal, indicating that both of the regions are derived from the left LPM expressing *Pitx2* (Figure 3, O and P).

### Pitx2 suppresses CCS formation.

**Figure 4:**
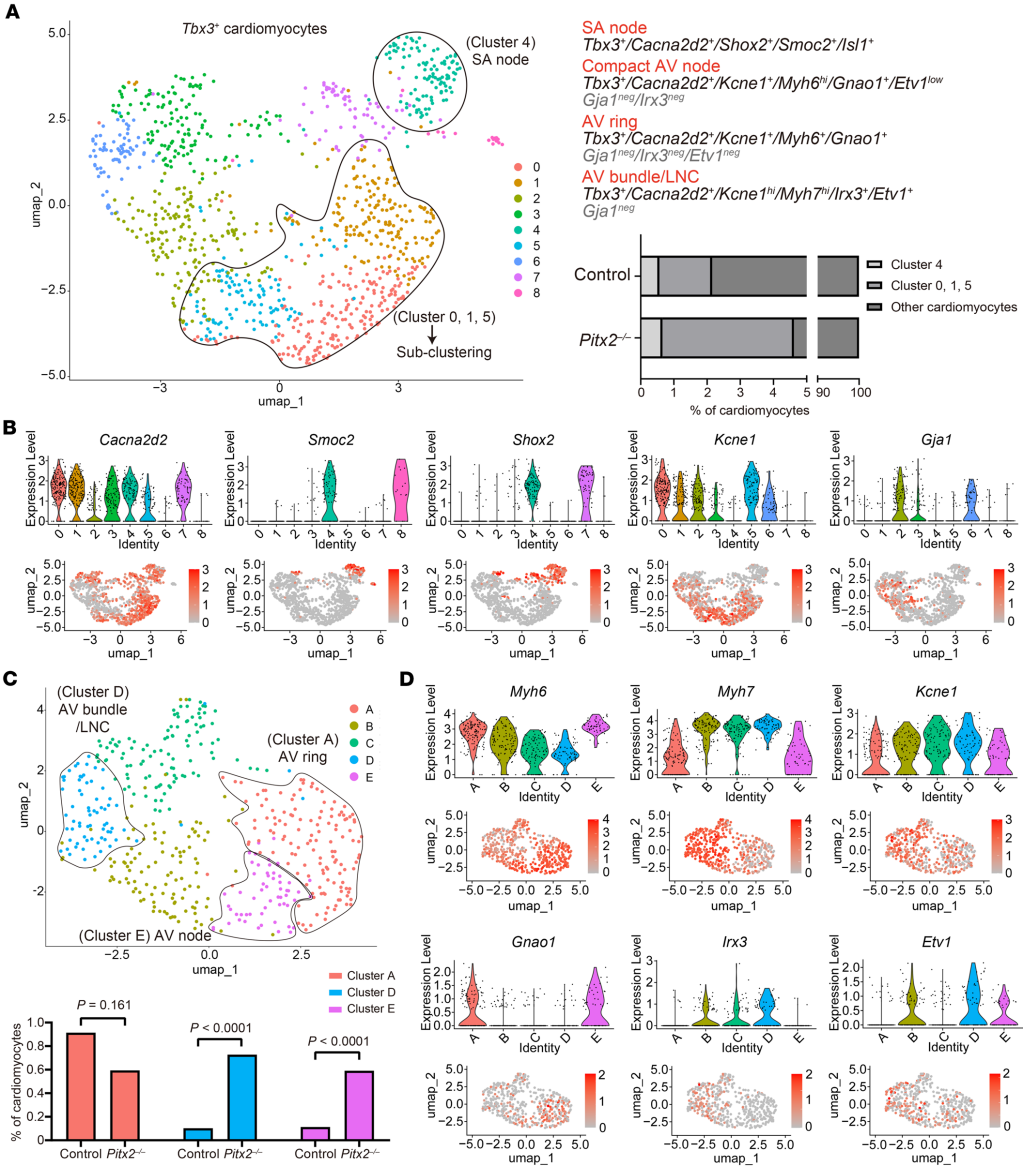
Altered cardiac conduction system population in *Pitx2*^–/–^ embryos. (**A**) UMAP visualization of *Tbx3*-expressing cardiomyocytes at E13.5 (control, 9739 cells; *Pitx2*^–/–^, 8247 cells). The graph shows the proportions of CCS-related cells among the cardiomyocytes: cluster 4 (control, 55 cells; *Pitx2^–/–^*, 54 cells); clusters 0, 1, and 5 (control, 155 cells; *Pitx2^–/–^*, 327 cells); and the remaining clusters (control, 9,529 cells; *Pitx2^–/–^*, 7,866 cells). (**B**) Expression patterns of *Cacna2d2*, *Smoc2*, *Shox2*, *Kcne1*, and *Gja1* visualized by feature and violin plots derived from the UMAP in **A**. (**C**) Subclustering analysis of clusters 0, 1, and 5 from **A**. The graph shows the ratios of subclusters among the cardiomyocytes: cluster A (control, 89 cells; *Pitx2^–/–^*, 49 cells); cluster D (control, 10 cells; *Pitx2^–/–^*, 60 cells); and cluster E (control, 11 cells; *Pitx2^–/–^*, 49 cells). *P* values were calculated using the Fisher’s exact test. (**D**) Feature and violin plots showing the expression of *Myh6*, *Myh7*, *Kcne1*, *Gnao1*, *Irx3*, and *Etv1* based on the UMAP analysis in **C**. LNC, lower nodal cells.

The abovementioned results indicated that CCS development is suppressed in regions where *Pitx2* is expressed. To confirm this, we analyzed published single-cell RNA-Seq (scRNA-Seq) data from control and *Pitx2*^–/–^ hearts at E13.5 (48). The uniform manifold approximation and projection (UMAP) analysis identified clusters consistent with those previously published (Supplemental Figure 7, A and B) (48). To enrich putative CCS cells, *Tbx3*-positive cardiomyocytes were selected and subclustered (Figure 4A and Supplemental Figure 7, A and C). Because *Tbx3*-positive cells include non-CCS tissues at this stage, CCS clusters were identified with reference to recent scRNA-Seq analyses of CCS at E16.5 (49, 50). Feature and violin plots of the marker genes revealed an SA node cluster (cluster 4, characterized by *Cacna2d2*^+^/*Shox2*^+^/*Smoc2*^+^/*Isl1*^+^) (Figure 4, A and B). Notably, the proportions of this cluster were similar between control and *Pitx2*^–/–^ hearts, suggesting that SA nodes bilaterally formed in *Pitx2*^–/–^ hearts may be reduced in size, as observed in the *Cryptic* mutant. Next, we focused on clusters (0, 1, and 5) defined by *Cacna2d2*^+^/*Kcne1*^+^/*Gja1*^neg^ (Figure 4, A and B), likely encompassing the compact AV node, nodal AV ring, and AV bundle (49). Subclustering yielded 5 new clusters (A–E) (Figure 4C). The expression profiles of clusters A (*Myh6*^+^/*Gnao1*^+^/*Etv1*^neg^) and E (*Myh6*^hi^/*Gnao1*^+^/*Etv1*^lo^) corresponded to those of the AV ring and compact AV node, respectively (Figure 4D) (49, 50). In *Pitx2*^–/–^ hearts, the proportion of cluster A decreased (control: 89/9,739 cells vs. *Pitx2*^–/–^: 49/8,247 cells), whereas that of cluster E increased (control: 11/9,739 cells vs. *Pitx2*^–/–^: 47/8,247 cells), suggesting the presence of dual AV nodes, as observed in *Cryptic*^–/–^ hearts. Cluster D, characterized by *Myh7*^hi^/*Irx3*^+^/*Etv1*^+^, likely represents the AV bundle/lower nodal cells (Figure 4D) (49). This population was significantly increased in *Pitx2*^–/–^ hearts (control: 10/9,739 cells vs. *Pitx2*^–/–^: 60/8,247 cells), indicative of the development of the anterior AV bundle. Clusters B and C did not correspond to the known CCS components at E16.5 (49), implying that these clusters represent an immature CCS or an abnormally developed population similar to that in the superior AV cushion of *Cryptic*^–/–^ embryos. Together, these results suggested that AVCS properties were altered in *Pitx2*^–/–^ embryos.

### Suppressed CCS development in Lefty1^–/–^ embryos.

**Figure 5:**
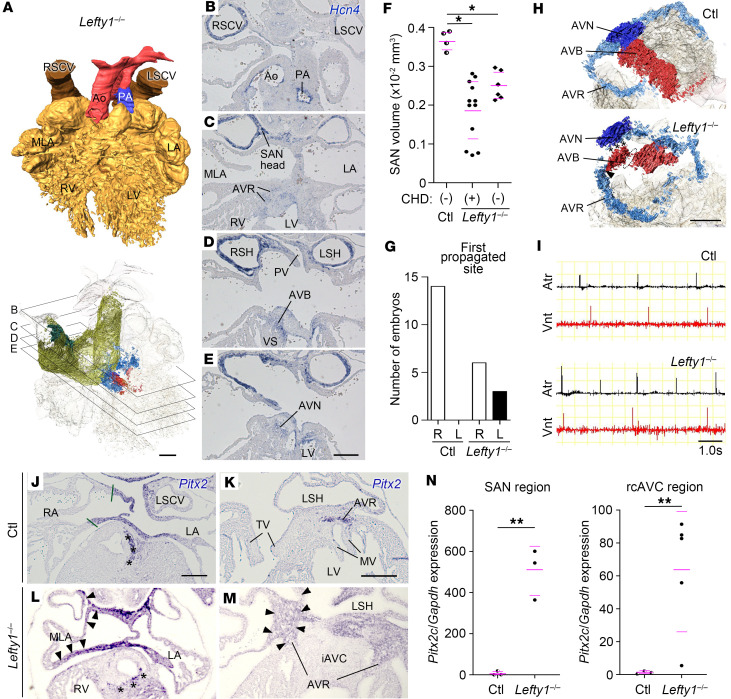
Hypoplastic sinoatrial node and atrioventricular block in *Lefty1*^–/–^ embryos. (**A**–**E**) Representative 3D-reconstructed hearts (**A**) and corresponding *Hcn4* in situ hybridization of a *Lefty1*^–/–^ embryo (*n* = 7) (**B**–**E**) at E14.5, as in Figure 1, A–E. (**F**) SA node head volumes in control (*Lefty1*^+/–^, Ctl) and *Lefty1*^–/–^ hearts at E12.5 (*n* = 4–12; biological replicates). The magenta bars denote mean ± SD. *P < 0.001 (unpaired 2-sided *t* test with Welch’s correction). (**G**) Atrial side where the action potential first propagated (*n* = 9–14). (**H**) 3D-reconstructed *Hcn4* expression in control and *Lefty1*^–/–^ hearts at E14.5 (different heart from **A**). The AV conduction systems are viewed from the right craniodorsal side. Asterisks and dotted areas: discontinuous *Hcn4* expression at the AV node/AV bundle junction and within the AV bundle, respectively. Arrowhead: continuous expression between the AV bundle and ring. (**I**) Action potentials in the atrium (Atr) and ventricle (Vnt) of control and *Lefty1*^–/–^ hearts at E12.5, traced using voltage mapping. (**J**–**M**) Transverse sections showing *Pitx2* expression in control (**J** and **K**) and *Lefty1*^–/–^ (**L** and **M**) hearts. Green bars indicate *Pitx2* expression boundary. Arrowheads indicate ectopic *Pitx2* expression. Asterisks mark *Pitx2* expression in the septal branch. Scale bars: 200 μm; bars in **E**, **J**, and **K** apply to **B**–**D**, **L**, and **M**, respectively. (**N**) RT–qPCR of *Pitx2c* in laser-microdissected heart tissues from control and *Lefty1*^–/–^ embryos at E12.5, quantified in the SA node region, including the adjacent superior caval vein and right atrial tissue, and in the right caudodorsal AV canal (rcAVC) region. The magenta bars indicate mean ± SD. **P < 0.05 (unpaired 2-sided *t* test with Welch’s correction). Abbreviations as in Figure 1 except for iAVC, inferior atrioventricular cushion; (L/R)SH, (left/right) sinus horn; MLA, morphologically left atrium.

The restricted expression of *Pitx2* in the heart suggests that appropriate assignment of *Pitx2* expression domains by the left-right axis is fundamental for CCS development. To examine the potential effect of altered *Pitx2* expression on CCS formation, we analyzed the CCS of *Lefty1*^–/–^ embryos using *Hcn4* ISH. *Lefty1*^–/–^ mice develop left isomerism by ectopically expressing *Pitx2* to varying degrees in the right LPM (26). Among the 7 *Lefty1*^–/–^ embryos examined at E14.5, 6 exhibited CHD accompanying left atrial isomerism, including AVSD (*n* = 5/7), DORV (*n* = 4/7), TGA (*n* = 1/7), and intracardiac TAPVC (*n* = 3/7) (Supplemental Table 1). In *Lefty1*^–/–^ embryos, the SA node was hypoplastic (E14.5: *n* = 6/7; E12.5: *n* = 11/18) (Figure 5, A–D), consistent with the characteristics of left isomerism in humans (34, 36, 37). The volumetry at E12.5 showed that the average SA node volume in *Lefty1*^–/–^ embryos was significantly reduced in comparison with that in control embryos (*Lefty1*^–/–^ with CHD: 1.87 ± 0.74 × 10^–3^ mm^3^ or *Lefty1*^–/–^ without CHD: 2.50 ± 0.33 × 10^–3^ mm^3^ vs. control: 3.67 ± 0.22 × 10^–3^ mm^3^) (Figure 5F). The volumes in *Lefty1*^–/–^ hearts with CHD varied, likely reflecting the degree of ectopic *Pitx2* expression (Figure 5N). Consistent with the hypoplastic SA node, the abnormal propagation of action potential in the atrium was noted in *Lefty1*^–/–^ embryos at E12.5. Although the difference of first breakthrough site was not significant between control and *Lefty1*^–/–^ embryos (control: right, *n* = 11/14; middle, *n* = 3/14 vs. *Lefty1*^–/–^: right, *n* = 5/9; middle or left, *n* = 4/9); *P* = 0.363 from Fisher’s exact test), the ratio that the left-sided atrium first propagated the action potential significantly increased in *Lefty1*^–/–^ embryos (control: right, *n* = 14/14 vs. *Lefty1*^–/–^: right, *n* = 6/9; left, *n* = 3/9; *P* = 0.0474 from Fisher’s exact test) (Figure 5G and Supplemental Figure 8).

In control embryos at E14.5 and E12.5, *Hcn4* expression in the AV node and bundle/IV ring was continuous (Figure 5H and Supplemental Figure 9). Notably, a discontinuity in *Hcn4* expression between the AV node and bundle/IV ring was observed in *Lefty1*^–**/**–^ hearts with CHD (*n* = 2/4 at E14.5; *n* = 4/9 at E12.5) (Figure 5H and Supplemental Figure 9), which was reminiscent of the dissociation of both components in left isomerism in humans (34, 35). In addition, the staining for *Hcn4* was weakened in the AV node of *Lefty1*^–/–^ embryos at E14.5 (*n* = 3/4) (Figure 5E). Because discontinuous *Hcn4* expression suggests the occurrence of AV block in *Lefty1*^–/–^ embryos, we analyzed successive action potential in the atrium and ventricle at E12.5 using voltage mapping. Among the 9 *Lefty1*^–/–^ embryos examined, we detected second-degree (*n* = 1/9) and third-degree (*n* = 1/9) AV blocks, whereas such blocks were not observed in control embryos (*n* = 12/12) (Figure 5I). To correlate the ectopic *Pitx2* expression with the CCS of *Lefty1*^–/–^ embryos, we examined the expression of *Pitx2*, *Hcn4*, and *Myl7* in adjacent sections at E14.5 (Figure 5, J–M, and Supplemental Figure 10). As expected, *Pitx2* was ectopically expressed in the hypoplastic SA node and around the entire AV orifice (*n* = 2/2) (Figure 5, L and M). In one case, *Hcn4* expression in the AV bundle was proximally terminated with a blind end, and its connection to the *Myl7*^+^ population, representing the compact AV node, was not observed (Supplemental Figure 10), indicating the disconnection of the AV bundle from the AV node. The ectopic expression of *Pitx2c* was further confirmed by RT-qPCR of the SA node region and the right caudodorsal region of the AV canal in *Lefty1*^–/–^ embryos at E12.5 (Figure 5N). Together, these results indicate that the left-right axis determines the disposition and function of the CCS, likely by appropriately defining the expression domains of *Pitx2* in the heart.

## Discussion

Nearly 120 years ago, Sunao Tawara discovered the AV node and the AV conduction pathway (51). The entire CCS was then found to be asymmetrically disposed along the body axes. Although such a disposition is often altered in patients with CHD (34, 52, 53), the underlying molecular mechanisms remain to be fully elucidated. This study showed that the left-right axis, established prior to overt heart tube formation, plays a decisive role in defining the disposition of the AV conduction axis and the SA node.

In this study, we utilized *Cryptic* and *Lefty1* mutants, defective in the left-right axis formation, as models of human heterotaxy to analyze hearts in which LPM-derived lineages had either lost or ectopically gained *Pitx2* expression. In WT hearts, the central CCS was formed in domains lacking *Pitx2* expression. Notably, the anterior AV node developed in association with reduced *Pitx2* expression in *Cryptic*^–/–^ embryos, whereas dissociation of the AV node and AV bundle occurred, accompanied by ectopic *Pitx2* expression in *Lefty1*^–/–^ embryos. Although Nodal signaling also controls cardiac looping independently of *Pitx2*, no evidence exists for *Cryptic* or *Lefty1* directly affecting the CCS development via *Pitx2*-independent mechanisms. Thus, although we cannot exclude the possibility that the CCS abnormalities in these mutants reflect additional, unidentified effects of Nodal signaling in the LPM, the scRNA-Seq analyses of *Pitx2* mutants support the interpretation that *Pitx2* suppresses AVCS development.

The development of each CCS component is regulated by distinct transcriptional networks, among which T-box transcription factors play pivotal roles (1, 2). Haplo-deficiency of mouse *Tbx5*, which functions upstream of these networks, causes defects in the formation of the ventricular conduction system and arrhythmias including AV block and SA pauses (54, 55). Tbx5 induces the expression of *Shox2* and *Tbx3* in the SA node (56, 57), as well as the expression of *Tbx3* in the AV node and AV bundle (16, 58). Based on studies on atrial fibrillation, *Pitx2* expression in the AV canal and IV ring may interfere with networks regulated by T-box transcription factors. In the left atrium, Pitx2 negatively regulates the expression of conduction genes driven by Tbx5 (23, 59). In cardiomyocytes of the pulmonary vein, Pitx2 likely interacts with a set of transcription factors, including the T-box family, to suppress gene expression (60). Furthermore, *Pitx2* induces the expression of microRNAs (*miR-17-92* and *miR-106b-25*) to repress the SA node program by directly targeting *Shox2* and *Tbx3* (22). *miR-17-92–*null mutants also upregulate *Tbx3* expression in the left AV canal (22). *Pitx2* may progressively shape the disposition of AVCS by downregulating and/or interfering with T-box genes in a manner different from that of the *Pitx2*/*Shox2* pathway in the SA node.

The AV canal comprises the descendants of the left and right LPM, the contributions of which are regionally specified. In mouse embryos, the left posterior second heart field contributes to the superior and left lateral AV canal, whereas the right posterior second heart field contributes to the inferior AV canal, which gives rise to the compact AV node (5, 47). The mode of *Pitx2c* transcriptional regulation and our *Pitx2 17-Cre* labeling further support the notion that the septal branch and the superior AV canal descend from the left LPM that received Nodal signaling (25, 29, 30). Although no direct evidence exists for the caudal IV ring/AV bundle deriving from the right LPM in mice, the right side of the linear heart tube in chick embryos contributes to the caudal portion of ventricles, suggesting the right-sided origin of the caudal IV ring (27). These observations suggest that the entire AVCS probably originates from right-sided LPM-derived precursor cells lacking *Pitx2c* expression.

**Figure 6:**
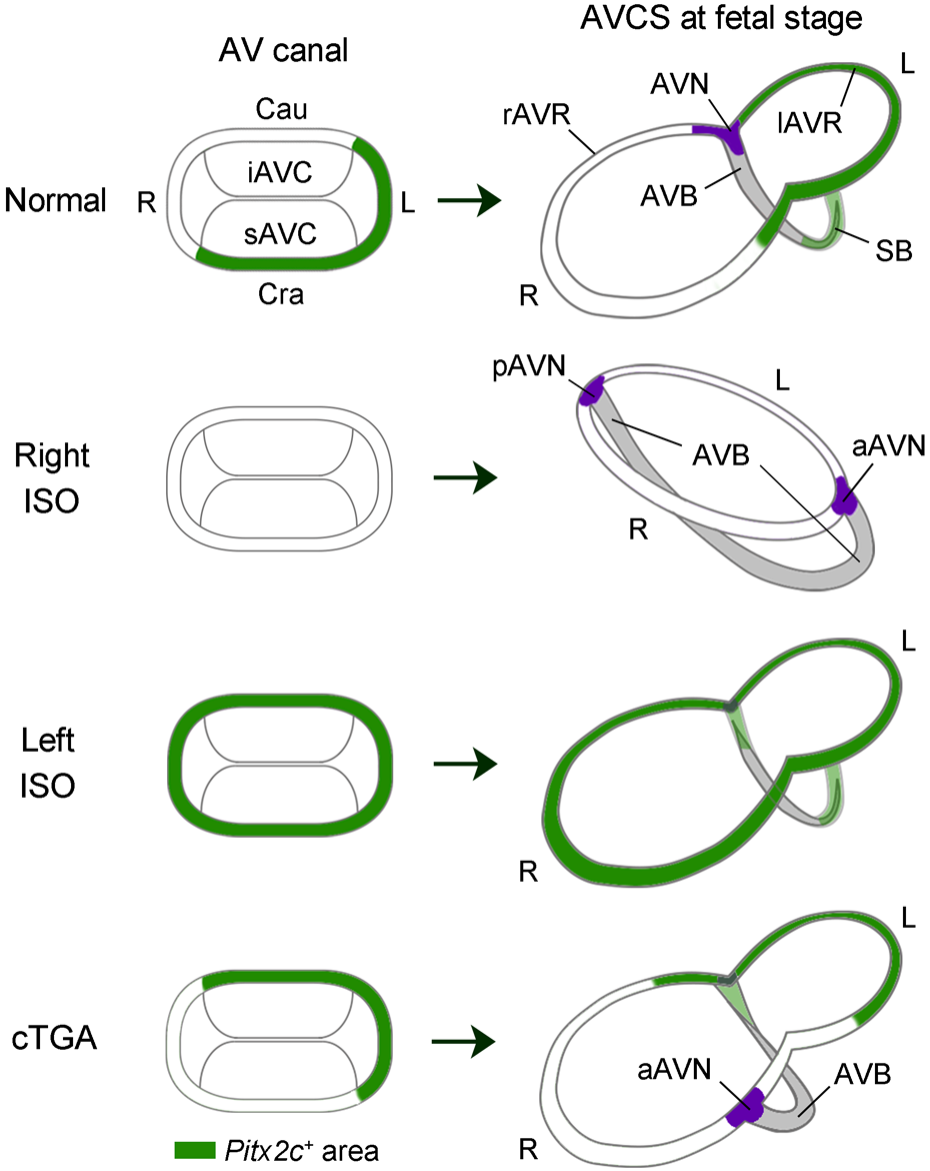
Model for the formation of atrioventricular conduction axis under normal and laterality-defective conditions. Schematic representations of the AV canal (left) and AVCS (right), derived from the AV canal and the IV ring under various conditions, are shown. Green areas denote the expression domain of *Pitx2c*. The connection between the AV node and AV bundle is established at the crosspoint(s) of the AV and IV rings, where *Pitx2c* expression is absent. The extent of loss and gain of *Pitx2c* expression in right and left atrial isomerism, respectively, varies depending on the underlying cause. In right isomerism, the AV rings with atrioventricular septal defect are depicted. In left isomerism, the most severe case — likely causing a hypoplastic AV node and AV dissociation in patients (35) — is shown. The corrected TGA model is based on the hypothesis that *Pitx2c*-expressing cells in the left LPM are inversely assigned to the AV canal and IV ring by reversed cardiac looping. AVB, atrioventricular bundle; (a/p)AVN, (anterior/posterior) atrioventricular node; Cau, caudal; Cra, cranial; cTGA, corrected transposition of the great arteries; (i/s)AVC, (inferior/superior) atrioventricular cushion; ISO, isomerism; (l/r)AVR, (left/right) atrioventricular ring; SB, septal branch.

Observations of human hearts with various CHDs underscore the importance of proper atrial and ventricular septal alignment for disposition of the AV conduction axis (52, 53). Because the IV septal ridge carries the AV bundle, displacement of the ventricular septum in CHD potentially alters the connection between the AV bundle and AV node (53). Additionally, in human heterotaxy, the AV conduction pathways are influenced by ventricular topology determined by the direction of cardiac looping (34). The region-specific expression of *Pitx2* in the AV canal and IV ring is most probably due to the conversion of the initial expression domain by cardiac looping, as was also reported in the ventricles of chick embryos (27). Given that Pitx2 suppresses the development of AVCS, the direction of cardiac looping would be a critical factor in determining the disposition and integrity of the AVCS. Here, we propose a unified model for the disposition of AVCS in CHD with laterality defects (Figure 6). In a normal heart, *Pitx2*-expression domains assigned by dextral looping suppress the development of the CCS to shape the AV conduction axis from the AV canal and IV ring. Right isomerism suggests the loss of *Pitx2* expression in the heart. Under this condition, dual AV nodes develop, irrespective of the looping direction (34). Formation of the anterior AV bundle and sling depends on the disappearance of *Pitx2* expression in the septal branch. Left isomerism suggests that *Pitx2* expression is induced bilaterally in the LPM. This varied ectopic expression likely accounts for the array of CCS abnormalities observed in patients with left isomerism (34, 35). AV dissociation would occur when dextral looping assigns descendant cells of the right LPM ectopically expressing *Pitx2* to the inferior AV canal/caudodorsal IV ring. When sinistral looping occurs with precursor cells of the AV canal and IV ring in the right LPM averting ectopic *Pitx2* expression, *Pitx2*-expressing cells in the left LPM would be assigned to the inferior AV canal but not to the superior AV canal/septal branch, thereby resulting in AV dissociation in the posterior region and allowing the development of the anterior AV node and anterior AV bundle (34). Finally, corrected TGA is characterized by a discordant AV connection. We speculate that this situation would occur if the heart tube loops independently of the left-right axis established by the *Nodal*/*Pitx2* pathway. In the hearts of the {S, L, L} segmental anatomy (situs solitus, L-looped ventricles, and leftward-positioned aorta), *Pitx2*-expressing cells in the left LPM would be assigned to the inferior AV canal and the caudodorsal IV ring, where *Pitx2* suppresses the development of CCS. Consequently, an anterior AV node devoid of *Pitx2* expression develops, to which the AV bundle connects, as observed in human corrected TGA (52).

In this study, we found that the left-right axis determines the disposition of the AVCS, most likely via *Pitx2* expression. The asymmetric expression of *Pitx2* in the heart is also required for ventricular remodeling, including that of the AV canal and IV septum (46). Thus, dysregulated *Pitx2* expression is expected to cause both structural defects and abnormalities in the disposition and integrity of the AVCS, reflecting its dual role in cardiac morphogenesis and development of the conduction system. Moreover, malformations of AV connection, even those independent of laterality defects, may also alter the final position of *Pitx2*-expressing domains. Evaluation of the *Pitx2* expression domains at the AV junction in such CHDs may provide important insights into the mechanisms underlying malformed AVCS.

## Methods

### Sex as a biological variable.

To our knowledge, there are no reports indicating a sex difference in the development of the CCS or its congenital anomalies. Therefore, we did not determine the sex of embryos analyzed in this study.

### Mice.

Embryos were obtained from the following sources: ICR mice (Japan SLC); mutant lines including *Cryptic* (backcrossed to FVB/N for more than 15 generations) (32) and *Lefty1* (B6/129 hybrid background) (26); and reporter lines (B6/129 hybrid background) including *Pitx2* *17*-*Cre* (a gift from H. Shiratori and H. Hamada, Osaka University) and *CAG-CAT-LacZ* (a gift from J. Miyazaki, Osaka University) (29, 61). Considering the varied phenotypes observed in *Cryptic* and *Lefty1* mutants, embryos were collected until a sufficient number of homozygous mutant embryos had been obtained to cover the full range of phenotypic variations in each experiment. Homozygous mutant embryos were analyzed concurrently with control embryos from the same genetic background. Noon on the day a vaginal plug was detected was designated as E0.5. Embryos were collected after euthanasia of pregnant females by cervical dislocation and fixed with 4% paraformaldehyde (PFA) in PBS or 1% PFA/0.2% glutaraldehyde in PBS containing 0.02% Nonidet P-40 (PBN) for X-gal staining. Genotypes were examined by PCR using tail genomic DNA, as described previously (26, 29, 32, 61).

### X-gal staining of CAG-CAT-LacZ embryos.

Fixed embryos were washed with PBN and stained with 40 mg/mL X-gal (5-bromo-4-chloro-3-indolyl-β-D-galactoside; Sigma-Aldrich) in PBS containing 4 mM potassium ferrocyanide, 4 mM potassium ferricyanide, and 1 mM MgCl_2_. Sections of stained embryos were counterstained with Eosin Y (Merck).

### In situ hybridization.

Serial paraffin sections (10 μm at E12.5; 12 μm at E14.5–18.5) were deparaffinized with Lemosol (Wako), treated with 2 μg/mL Proteinase K (Nakalai), and refixed with 4% PFA in PBS. After acetylation with 0.25 M acetic anhydride and 0.1 M triethanolamine (Sigma-Aldrich), the sections were hybridized overnight with a digoxigenin-labeled probe. Excess probe was removed by saline sodium citrate washes and 10 μg/mL RNase A treatment. After blocking with 10% sheep serum, the sections were incubated with anti-digoxigenin antibody conjugated to alkaline phosphatase (11093274910, Roche), washed with Tris–buffered saline containing 0.1% Tween 20 (Nakalai), and developed with nitro blue tetrazolium and 5-bromo-4-chloro-3-indolyl-phosphate (Roche). After treatment with DAPI, the sections were mounted with 80% glycerol or Softmount (Wako). Bright-field and fluorescence images were captured using an upright fluorescence microscope (Leica DM5000B). The probes used in this study included *Hcn4*, *Tbx3*, *Gja5*, *Pitx2* (all isoforms), *Shox2*, and *Myl7*. The plasmid for *Tbx3* and *Shox2* was provided by H. Kokubo (Tohto University), and plasmids for *Gja5* and *Myl7* were provided by J. Takeuchi (Institute of Science Tokyo). For *Hcn4*, DNA fragments corresponding to 657–1265 and 2908–4437 of NM_001081192.2 were cloned, and antisense riboprobes were mixed at a 1:1 ratio.

### 3D reconstruction.

Image sets of *Hcn4*-staining and DAPI fluorescence of serial heart sections were processed with Amira Software (Visualization Science Group). *Hcn4* images were aligned to DAPI images with the AlineSlices command, converted to grayscale with a red color channel using the CastField module, and inverted using the Arithmetic module. *HCN4*-positive regions were manually traced and color-coded for each CCS component, and 3D reconstruction was performed using the SurfaceGen command. For standardizing SA node head volume measurement, voxels with intensities above 100 were labeled, and their mean intensity was calculated; voxels exceeding 75% of this mean were then relabeled. The labeled field was 3D surface-reconstructed using the unconstrained smoothing process of SurfaceGen, and the volume was measured with the MaterialStatistics module. The cardiovascular lumen was labeled by selecting voxels with intensities above 210 in DAPI images and reconstructed similarly.

### IHC.

Deparaffinized sections (12 μm) of E18.5 hearts were subjected to antigen retrieval by heating in citrate buffer (pH 6.0) at 120°C for 5 minutes. After blocking with Blocking One-P (Nakalai), the sections were incubated sequentially with anti-HCN4 antibody (APC-052, Alomone Labs) and Alexa Fluor 488 anti-rabbit antibody (A-11094, Invitrogen). After counterstaining with DAPI, the images were obtained with a confocal laser scanning microscope (Leica SP8).

### RT-qPCR analysis.

Serial paraffin-embedded sections (12 μm) of E12.5 hearts were mounted on PEN-membrane slides (11505189, Leica). After deparaffinization, specific regions of interest were separately collected by laser microdissection (Leica LMD6500). Total RNA from the collected tissues was extracted using the RNeasy FFPE kit (QIAGEN) and reverse-transcribed with PrimeScript reverse transcriptase (Takara). qPCR was performed using Luna Universal qPCR Master Mix (New England Biolabs) on an Applied Biosystems QuantStudio 3 system (Thermo Fisher Scientific). *Pitx2c* expression was normalized to *Gapdh* expression. The primer pairs used were as follows: (*Pitx2c*) 5′-CACCATCCCCAGGCGTTAG-3′ and 5′-GCCCTTATCTTTCTCTGCGAC-3′; (*Gapdh*) 5′-ACAGTCCATGCCATGCC-3′ and 5′-GCCTGCTTCACCACCTTCTT-3′.

### Analysis of scRNA-Seq data.

scRNA-Seq data from E13.5 *Pitx2 hd^–/–^* heart tissue, previously published by Hill et al. (48), were analyzed. The processed matrix files (GSE131181_e13.5.meta.data.csv.gz and GSE131181_e13.5.raw.data.csv.gz) were imported into Seurat (v 5.1.0; https://satijalab.org/seurat/). Quality control involved filtering out cells with fewer than 200 detected genes and excluding cells, including cardiomyocytes, with greater than 25% mitochondrial gene content, a threshold set according to sample characteristics. Potential doublets were identified and removed using the scDblFinder package (version 1.16.0; https://github.com/plger/scDblFinder; branch: master; commit ID: 99b947a49a4f3f3bd1a2203fe2296a6dc143fe11).. The filtered dataset was log-normalized. Principal component analysis was performed, and the top 30 principal components were used for UMAP and graph-based clustering. Differential gene expression between clusters was determined using the FindAllMarkers function, applying a nonparametric Wilcoxon rank-sum test with a log_2_ fold-change threshold of 0.5 and an FDR of 0.01. Marker genes were used to assign the cell types to each cluster. *Tbx3*-expressing cells in cardiomyocyte clusters were selected using the WhichCells function, followed by subclustering for further CCS analysis.

### Cardiac voltage mapping.

Embryos at E12.5 were collected in Tyrode’s solution at 37°C, and hearts were excised to preserve tissues adjacent to the superior vena cava. The isolated hearts were incubated in Tyrode’s solution at 37°C for 1 hour and then treated with 4 μM Di-4-ANEPPS (Invitrogen) at 37°C for 5 minutes. The hearts were placed in a dish with Tyrode’s solution at 37°C under a THT-macroscope (SciMedia), and Di-4-ANEPPS fluorescence, excited by a 150 W halogen light source (HL151, Moritex Corporation), was captured using a CMOS camera (MiCAM02, SciMedia) with 1.20 ms/frame (833 Hz) rate of 4096 frames. The acquired images were analyzed using BrainVision Analyzer software. Considering the variability in heart rates recorded, we analyzed the final beat recorded for each heart to determine the first breakthrough site and the propagation of the action potential. Hearts exhibiting high fluorescent noise or physical damage were excluded from the analysis.

### Statistics.

Statistical analyses were conducted using GraphPad Prism 8 software, with a *P* value less than 0.05 considered statistically significant. The statistical methods are described in the Results or in the figure legends and include Fisher’s exact test, 1-way ANOVA followed by Dunnett’s multiple-comparison test, and unpaired 2-sided *t* tests with Welch’s correction.

### Study approval.

All animal experiments were approved by the IACUC of Kyushu University, in accordance with the *Guide for the Care and Use of Laboratory Animals* (National Academies Press, 2011).

### Data availability.

The reanalyzed sequencing dataset is publicly available from NCBI’s Gene Expression Omnibus under accession number GSE131181 (48). Data generated and analyzed in this study are provided in the Supporting Data Values file.

## Author contributions

CM was responsible for conceptualization. SO and CM were responsible for the methodology. KJ, KK, KY, RM, and CM conducted the investigation. MS provided resources. KJ, RM, and CM conducted the formal analysis. CM performed validation. KJ, RM, and CM performed visualization. KJ, RM, and CM curated the data. AS and RT supervised the study. CM was responsible for project administration. CM wrote the original draft. KJ, RM, and CM reviewed and edited the manuscript. The order of the co–first authors was determined based on overall contributions to the study. KJ made major contributions to the original study through extensive wet-lab experiments, while RM performed the primary data analyses. During the revision process, RM played a central role in conducting additional wet-lab experiments requested by the reviewers. Both authors contributed substantially to the work, and the authorship order was determined by mutual agreement.

## Funding support

- Japan Society for the Promotion of Science KAKENHI grants (JP17H01571 and JP25K02625 to CM and JP25K11130 to KK).
- Naito Foundation (to CM).

## Supplementary Material

Supplemental data

Supporting data values

## Version 1 02/24/2026

In-Press Preview

## Version 2 03/23/2026

Electronic publication
