# Supplementary material for: Cardiac conduction system malformations in heterotaxy result from dysregulated Pitx2 expression
Source: JCI Insight. 2026 Feb 24;11(6):e199072. doi: 10.1172/jci.insight.199072 (PMC13043103; doi:10.1172/jci.insight.199072)
Supplement: Supplemental data [file jciinsight-11-199072-s242.pdf]

	Cryptic$^{-/-}$ (E14.5, *n* = 8; E18.5, *n* = 7)		*Lefty1*$^{-/-}$ (E14.5, *n* = 7)	
		(*n*)		(*n*)
Cardiac position	Levocardia	13	Mesocardia	6
	Dextrocardia	2		
Atrial arrangement	Right isomerism	15	Left isomerism	6
Atrial septum	Absent	15	Deficient/absent	5
Pulmonary veins	Infracardiac	8	To left-sided atrium	1
	To the midline of CA	7	To right-sided atrium	2
			To the midline of atrium	2
			To SV	1
Systemic veins (SCV)	Each SCV to ipsilateral side of atrium with venous valves	15	Each SCV to ipsilateral side of atrium with venous valves	1
			Left SCV to SV positioned in the midline	5
			Right SCV to right-sided atrium with or without short venous valves	5
Mode of atrioventricular connection	Common AV valve	13	Two AV valves	1
	Common AV valve with unilateral AVV stenosis/atresia	2	Common AV valve with unilateral AVV stenosis	4
			Common AV valve	1
Ventricular morphology	Complete AVSD with biventricular connection	12	Complete AVSD with biventricular connection	1
	Incomplete AVSD with biventricular connection	1	Complete AVSD with rudimentary RV	3
	DILV with rudimentary RV	2	DIRV with rudimentary LV	1
Ventricular septum	Perimembranous inlet VSD	7	Perimembranous inlet VSD	2
	Perimembranous outlet VSD	5	Perimembranous outlet VSD	4
	Intact ventricular septum	3		
Venricular topology	Right-handed	15	Right-handed	6
Ventriculo-arterial connections	d-TGA	11	Concordant	1
	DORV	4	d-TGA	1
			DORV	4

Supplemental Table 1. Summary of cardiac morphological characteristics in *Cryptic*$^{-/-}$ and *Lefty1*$^{-/-}$ embryos at E14.5 and E18.5. Only *Lefty1*$^{-/-}$ hearts with congenital heart defects were analyzed. AVV, atrioventricular valve; AVSD, atrioventricular septal defect; CA, common atrium; DILV, double inlet left ventricle; DIRV, double inlet right ventricle; DORV, double outlet right ventricle; d-TGA, dextro-transposition of the great arteries; LV, left ventricle; RV, right ventricle; SCV, superior caval vein; SV, sinus venosus; VSD, ventricular septal defect.

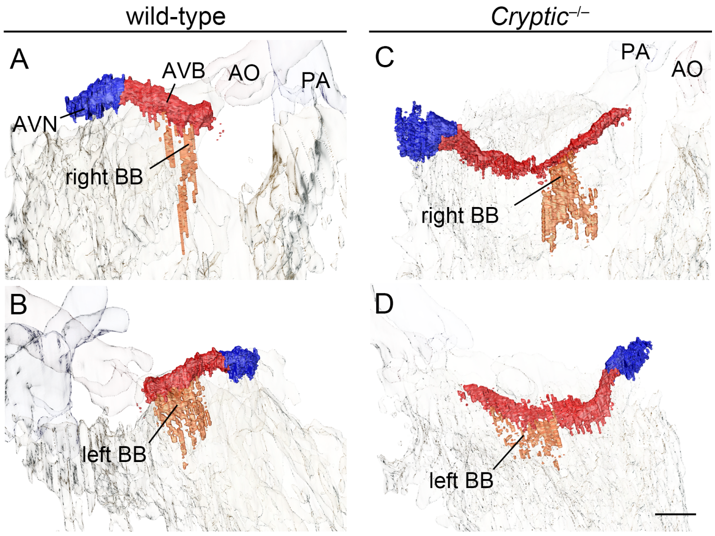

Supplemental Figure 1. Morphology of the right and left bundle branches in wild-type and *Cryptic*$^{-/-}$ hearts. The AV bundle and bundle branches in wild-type (**A** and **B**) and *Cryptic*$^{-/-}$ (**C** and **D)** hearts at E18.5 are shown based on *Hcn4* expression traces. Panels **A** and **C** show views from the right ventricular cavity, whereas panels **B** and **D** show views from left ventricular cavity. Scale bar, 200 µm. AO, aorta; AVB, atrioventricular bundle; AVN, atrioventricular node; BB, bundle branch; PA, pulmonary artery.

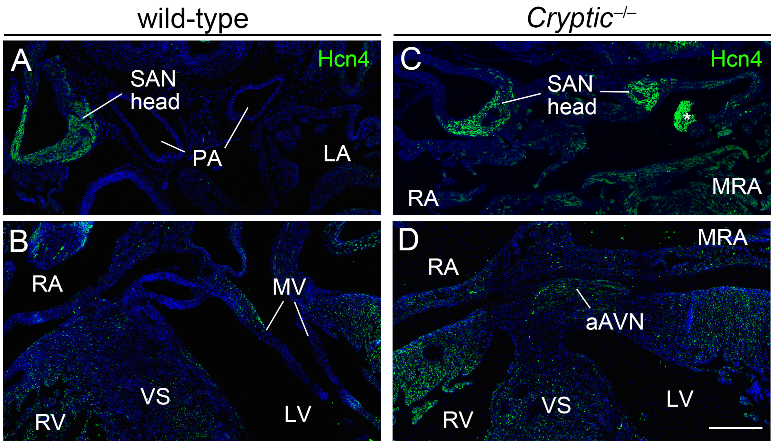

Supplemental Figure 2. Localization of Hcn4 in the sinoatrial (SA) nodes and the anterior atrioventricular (AV) node of the *Cryptic*$^{-/-}$ heart at E18.5. Hcn4 was detected by immunofluorescence in the hearts of wild-type (**A** and **B**) and *Cryptic*$^{-/-}$ (**C** and **D**) embryos. Transverse sections at the levels of the SA node (**A** and **C**) and the cranial AV junction (**B** and **D**) are shown. Bilateral SA nodes and the anterior AV node were observed in the *Cryptic*$^{-/-}$ heart. The asterisk indicates erythrocyte autofluorescence. Scale bar, 200 µm. aAVN, anterior atrioventricular node; (M)RA, (morphologically) right atrium; LA, left atrium; LV, left ventricle; MV, mitral valve; PA, pulmonary artery; RV, right ventricle; SAN, sinoatrial node; VS, ventricular septum.

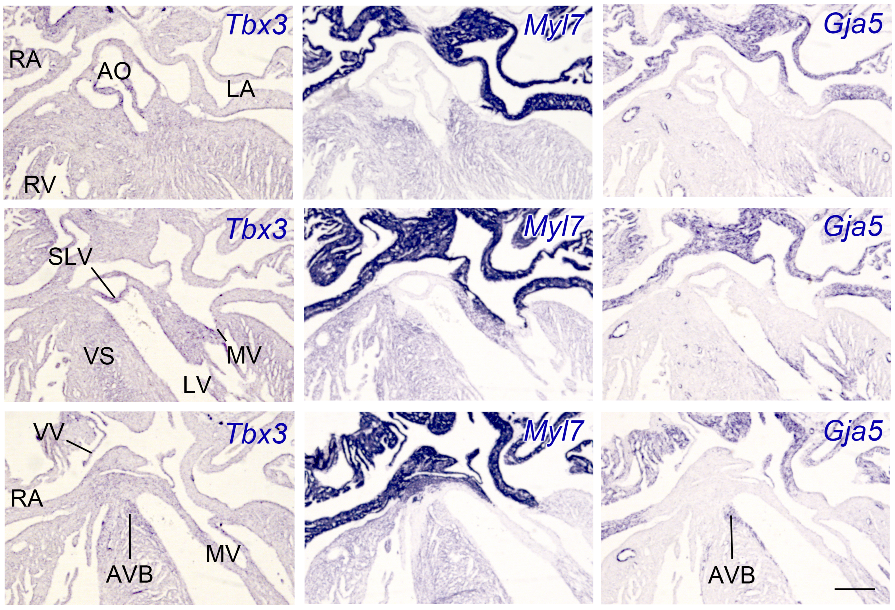

Supplemental Figure 3. Expression of the cardiac conduction system (CCS)-related genes around the cranial atrioventricular (AV) junction. Adjacent transverse sections of the heart from a wild-type embryo at E18.5 showing the expression of *Tbx3*, *Myl7*, and *Gja5*. The anterior AV node and AV bundle do not exist at the cranial AV junction. Scale bar, 200 µm. AO, aorta; AVB, atrioventricular bundle; LA, left atrium; LV, left ventricle; MV, mitral valve; RA, right atrium; RV, right ventricle; SLV, semilunar valve; VS, ventricular septum; VV, venous valve.

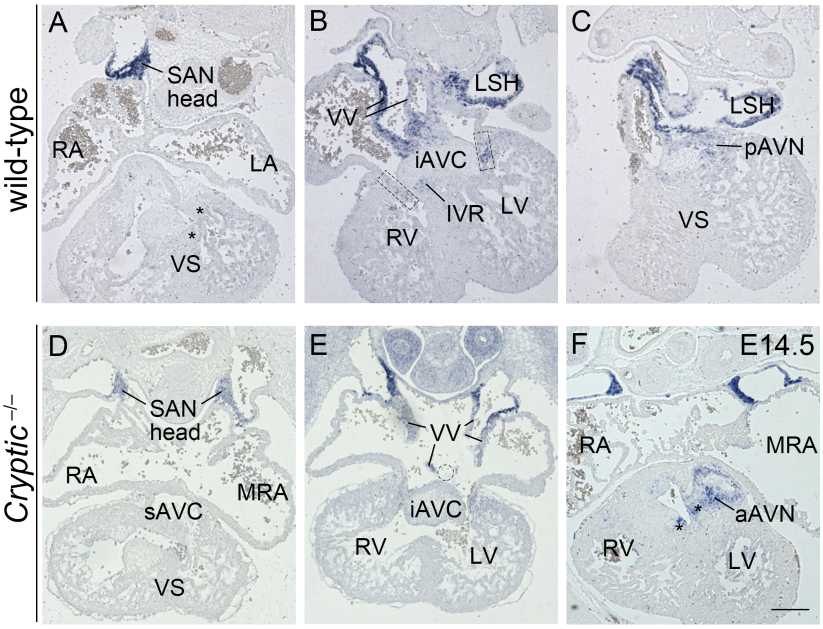

Supplemental Figure 4. *Hcn4* expression in the hearts of wild-type and *Cryptic*$^{-/-}$ embryos. Transverse sections showing *Hcn4* expression in the hearts of wild-type (**A**–**C**) and *Cryptic*$^{-/-}$ (**D**–**F**) embryos at E12.5 (**A**–**E**) and E14.5 (**F**). The asterisks in **A** and **F** show the route of the septal branch and *Hcn4*$^{+}$ anterior atrioventricular (AV) bundle, respectively. The dashed rectangles in **B** indicate the expression in the AV rings and AV valves. The dashed circle in **E** indicates the loss of dorsal mesenchymal protrusion (DMP)-derived tissue. Scale bar, 200 μm. aAVN, anterior atrioventricular node; (i/s)AVC, (inferior/superior) atrioventricular canal; IVR, interventricular ring; LA, left atrium; LSH, left sinus horn; LV, left ventricle; (M)RA, (morphologically) right atrium; pAVN, prospective atrioventricular node; RV, right ventricle; SAN, sinoatrial node; VS, ventricular septum; VV, venous valve.

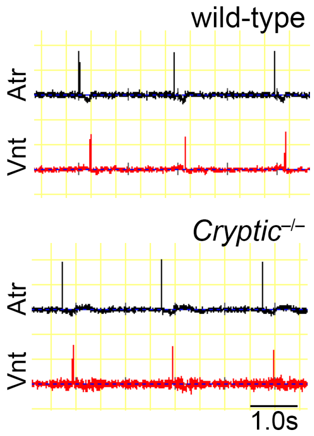

Supplemental Figure 5. Atrial and ventricular action potentials in wild-type and *Cryptic*$^{-/-}$ hearts at E12.5. Successive atrial (Atr) and ventricular (Vnt) action potential traces were recorded by voltage mapping. Representative traces from wild-type (n = 8) and *Cryptic*$^{-/-}$ (n = 9) embryos are shown.

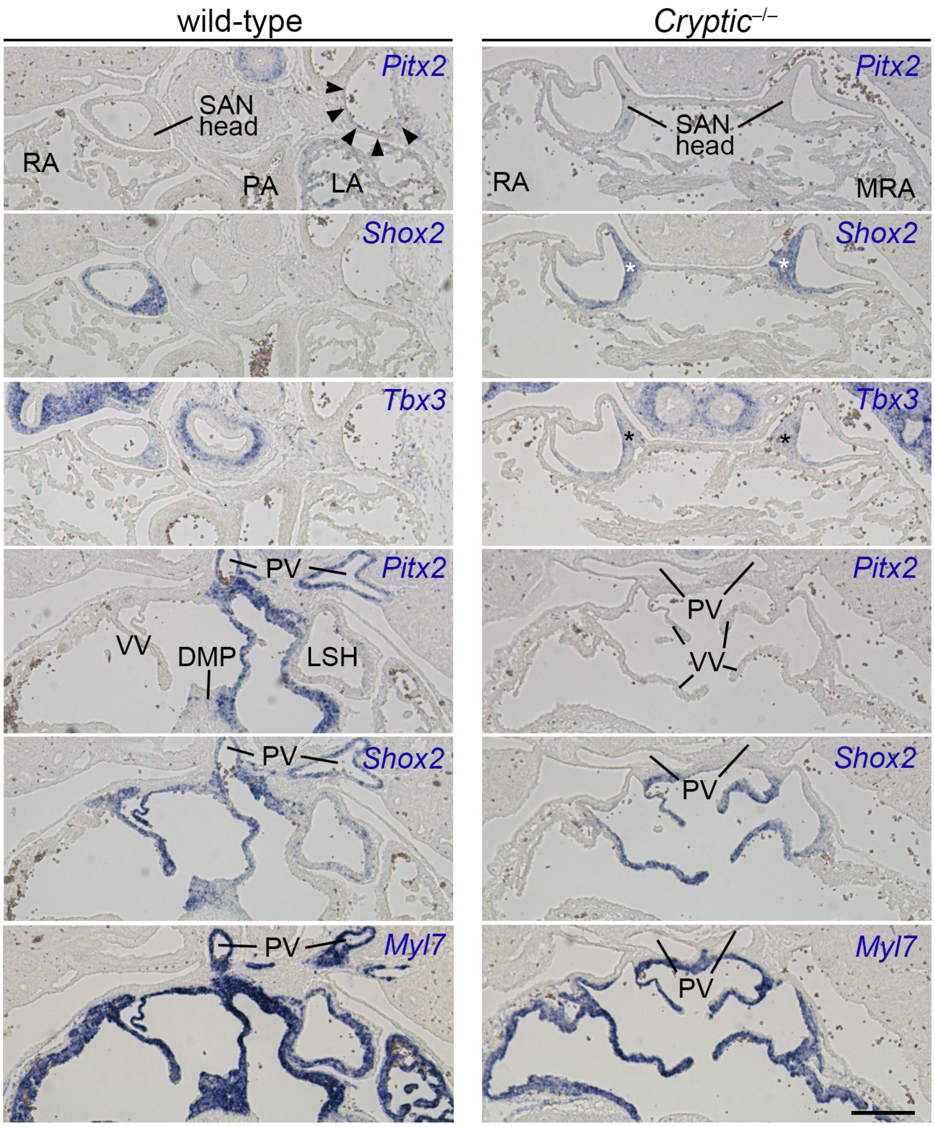

Supplemental Figure 6. Comparison of the expression domains of *Pitx2* and the cardiac conduction system (CCS)-related genes. Adjacent transverse sections of hearts from wild-type and *Cryptic*$^{-/-}$ embryos at E14.5 showing the expression of *Pitx2*, *Shox2*, *Tbx3*, and *Myl7*. The arrows indicate weak expression of *Pitx2* around the left superior caval vein. The asterisks indicate the expression of *Shox2* and *Tbx3* in bilaterally formed sinoatrial (SA) nodes in *Cryptic*$^{-/-}$ embryos. Note the loss of *Pitx2*, *Shox2*, and *Myl7* expression in the pulmonary veins of *Cryptic*$^{-/-}$ embryos. Scale bar, 200 µm. DMP, dorsal mesenchymal protrusion-derived tissue; LA, left atrium; LSH, left sinus horn; (M)RA, (morphologically) right atrium; PV, pulmonary vein; SAN, sinoatrial node; VV, venous valve.

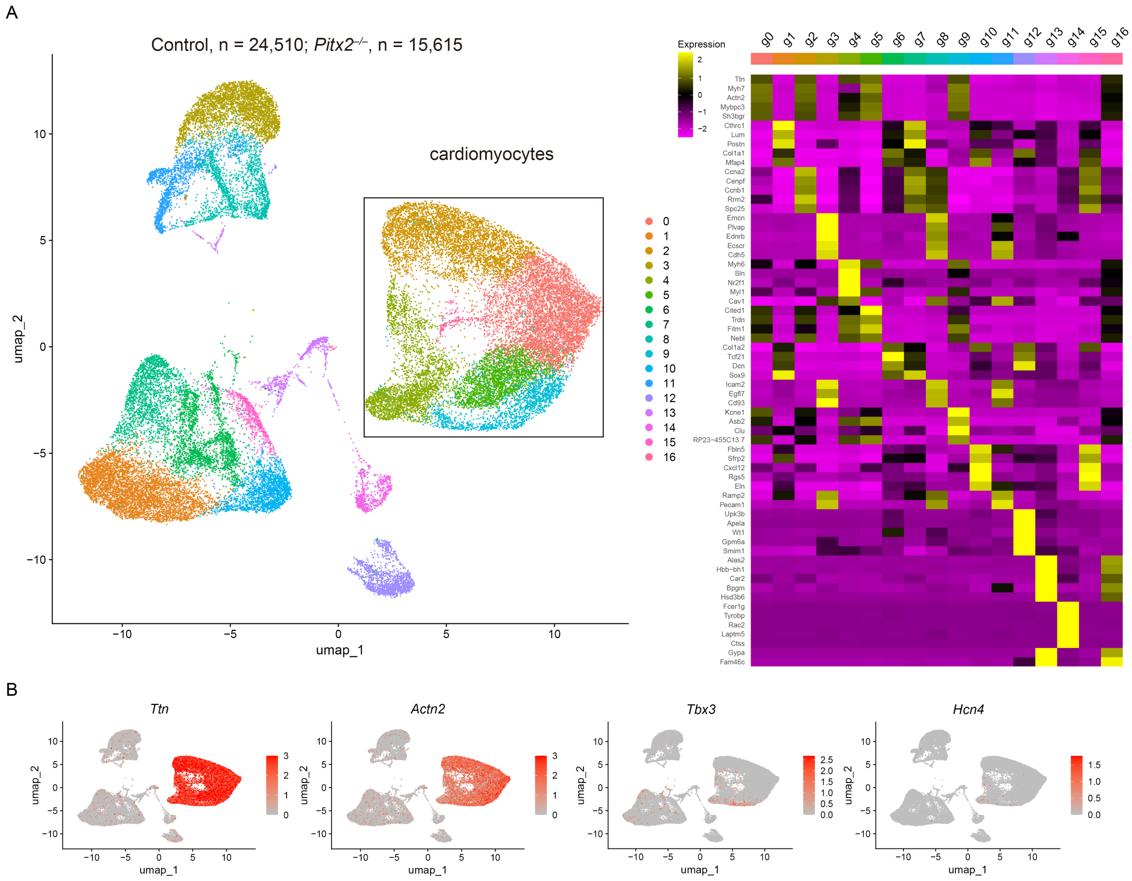

Supplemental Figure 7. Uniform Manifold Approximation and Projection (UMAP) analysis of scRNA-seq data for hearts from control and *Pitx2*$^{-/-}$ embryos at E13.5. (**A**) UMAP visualization of published scRNA-seq data (GSE131181_e13.5: Hill et al.). Following quality control and removal of doublets and lung cells, a total of 40,125 cells (control, *n* = 24,510; *Pitx2*$^{-/-}$, *n* = 15,615) was analyzed. Cardiomyocyte clusters are highlighted with a rectangle. (**B**) Differentially expressed genes for each cluster. (**C**) Feature plots showing the expression of *Ttn*, *Actn2*, *Tbx3*, and *Hcn4*.

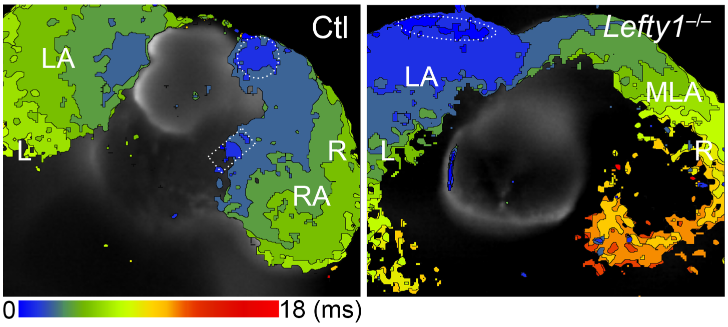

Supplemental Figure 8. Propagation of action potentials in control and *Lefty1*$^{-/-}$ hearts at E12.5. Representative voltage-mapping images obtained with Di-4-ANEPPS in control (*Lefty1*$^{+/-}$) (n = 14) and *Lefty1*$^{-/-}$ (n = 9) hearts at E12.5 (dorsal views). The dotted circles and rectangles indicate the sites where the action potential first appeared, around the head and tail of the SA node, respectively. (M)LA, (morphologically) left atrium; L, left; R, right; RA, right atrium.

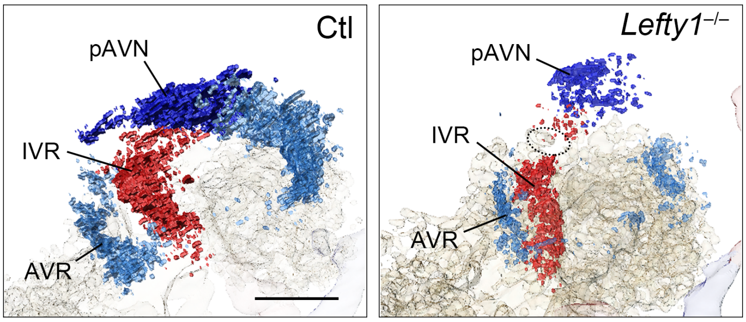

Supplemental Figure 9. Discontinuous *Hcn4* expression within the interventricular (IV) ring of a *Lefty1*$^{-/-}$ heart at E12.5. *Hcn4*-expressing domains were 3D-reconstructed in control (*Lefty1*$^{+/-}$) and *Lefty1*$^{-/-}$ hearts at E12.5. The atrioventricular (AV) conduction system was viewed from the right craniodorsal side. The dotted area marks a discontinuity in *Hcn4* expression within the caudodorsal IV ring, a region contributing to the developing AV bundle. AVR, atrioventricular ring; IVR, interventricular ring; pAVN, prospective atrioventricular node.

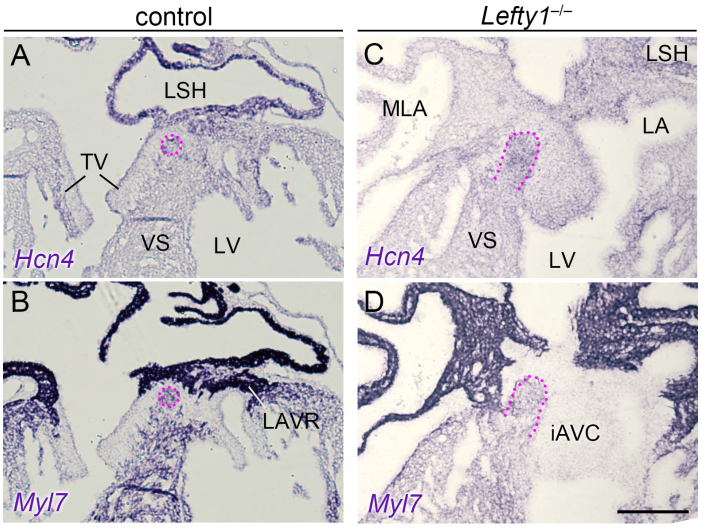

Supplemental Figure 10. Proximal region of the atrioventricular (AV) bundle in control and *Lefty1*$^{-/-}$ hearts at E14.5. Transverse sections of control (*Lefty1*$^{+/-}$) (**A** and **B**) and *Lefty1*$^{-/-}$ hearts (**C** and **D**) showing the expression of *Hcn4* (**A** and **C**) and *Myl7* (**B** and **D**). Panels **A** and **B** and panels **C** and **D** represent adjacent sections corresponding to Figure 5G and Figure 5I, respectively. Dotted areas indicate the AV bundle. Scale bar, 200 μm. iAVC, inferior atrioventricular cushion; LA, left atrium; LAVR, left atrioventricular ring; LSH, left sinus horn; LV, left ventricle; MLA, morphologically left atrium; TV, tricuspid valve; VS, ventricular septum.
